# LAMINA: a tool for rapid quantification of leaf size and shape parameters

**DOI:** 10.1186/1471-2229-8-82

**Published:** 2008-07-22

**Authors:** Max Bylesjö, Vincent Segura, Raju Y Soolanayakanahally, Anne M Rae, Johan Trygg, Petter Gustafsson, Stefan Jansson, Nathaniel R Street

**Affiliations:** 1 Research Group for Chemometrics, Department of Chemistry, Umeå University, SE-901 87 Umeå, Sweden; 2 CNAP *Artemisia *Research Project, Centre for Novel Agricultural Products, Department of Biology, PO Box 373, York, YO10 5YW, UK; 3 Department of Forest Sciences, 2424, Main Mall, University of British Columbia, Vancouver, BC, V6T 1Z4, Canada; 4 Umeå Plant Science Centre, Department of Plant Physiology, Umeå University, SE-901 87 Umeå, Sweden

## Abstract

**Background:**

An increased understanding of leaf area development is important in a number of fields: in food and non-food crops, for example short rotation forestry as a biofuels feedstock, leaf area is intricately linked to biomass productivity; in paleontology leaf shape characteristics are used to reconstruct paleoclimate history. Such fields require measurement of large collections of leaves, with resulting conclusions being highly influenced by the accuracy of the phenotypic measurement process.

**Results:**

We have developed LAMINA (Leaf shApe deterMINAtion), a new tool for the automated analysis of images of leaves. LAMINA has been designed to provide classical indicators of leaf shape (blade dimensions) and size (area), which are typically required for correlation analysis to biomass productivity, as well as measures that indicate asymmetry in leaf shape, leaf serration traits, and measures of herbivory damage (missing leaf area). In order to allow Principal Component Analysis (PCA) to be performed, the location of a chosen number of equally spaced boundary coordinates can optionally be returned.

**Conclusion:**

We demonstrate the use of the software on a set of 500 scanned images, each containing multiple leaves, collected from a common garden experiment containing 116 clones of *Populus tremula *(European trembling aspen) that are being used for association mapping, as well as examples of leaves from other species. We show that the software provides an efficient and accurate means of analysing leaf area in large datasets in an automated or semi-automated work flow.

## Background

Leaves are of fundamental importance to plants, representing the power generation facility and aerial environmental sensing units of plants, and by extension ultimately provide the energy for sustaining most terrestrial species on earth. A number of genes known to affect meristomatic pattern formation (e.g. *AS1 *and *WUS*, *KNOX *and *CLV*, see [1] for a review of leaf development), the rate of leaf primordia initiation [2] and that contribute to the determination of leaf length (*ROT3 *[3], *LNG *[4]) and width (*AN *[5]) have now been identified: less is known about the determination of leaf size currently. Despite these advances, it remains clear that leaf area development is a highly complex process that is influenced by genetic, hormonal and environmental factors. Quantitative Trait Loci (QTL) mapping of leaf development and leaf size and shape indicators suggests that these traits are under the control of many genes [6-15], with relatively few genes identified to date [1]. To advance the current understanding of leaf area development and final dimension determination requires the ability to phenotype large collections of leaves from QTL mapping populations, natural populations and forward genetic screens to identify and quantify loci/mutations influencing leaf characteristics. As well as being important to the fields of genetics, physiology, plant breeding and developmental biology, leaf shape parameters are also important as a means of reconstructing historical paleoclimate conditions [16,17], where information on leaf serration (depth and presence/absence) is used to accurately reconstruct past mean annual temperature [18,19]. Leaf size and shape parameters (physiognomy) were initially quantified using gridded paper, where a count of squares was used to measure leaf size, or through the development of allometric relationships between length, width and area, with length typically being measured and later used to calculate area using a regression model. This approach can work well within a single species but works poorly when applied to mapping populations, where segregation can lead to extensive variation in both leaf area and shape traits. It is equally inappropriate for forward genetic screens to identify leaf phenotypes, where the induced phenotypic changes are unpredictable. For many species, field-portable leaf scanning equipment can be used to measure leaf area and blade dimensions. However, such equipment cannot be used on large leaves and works poorly on species such as *Arabidopsis thaliana *due to small leaf area and the proximity of leaves to the soil. Such equipment is also often limited in the range of measurements provided and, as no digital image is captured, retrospective re-analysis using, for example, new software tools is not possible. More recently, methods have concentrated on the capturing of digital images of leaves (or fossils) with subsequent analysis using digital image analysis tools. A number of such tools already exist, but none of the currently available software was able to fulfil our needs. ImageJ [20] is a widely used application for the analysis of biological images and can be used to analyse area and blade dimensions. However, automated analysis is hard to achieve, as is the simultaneous measurement of area and blade dimensions when leaves are not square within the image. ImageJ offers no method to quantify leaf serration. The development of tools for measuring leaf area was reported in [21] and [22], however they offer little to extend the capabilities of ImageJ. More recently [23] reported the development of LeafAnalyser, which is an excellent tool to facilitate PCA analysis of leaf shape parameters. However, this tool does not report the type of dimensions that are typically required by plant breeders, physiologists, geneticists or palaeontologists and the software was not released as open source, negating the possibility of further development by the community. We additionally found that the implemented thresholding frequently required per-image manual adjustment, making the automated, rapid analysis of leaves more time consuming. We were interested in measuring basic leaf dimension parameters (area, length, width) as well as measures of leaf shape, symmetry, serration number and depth and the missing area within a leaf (as a measure of damage by biting herbivores) in a collection of naturally occurring clones of *Populus tremula*, the Swedish Aspen (SwAsp) collection, that are being grown in common garden experiments in the south and north of Sweden [24] and that are being used for association mapping [25]. This species has well defined, characteristic leaf serrations that we had visually observed to show variation between clones within the SwAsp collection. We were therefore interested to see to what degree leaf serration was under genetic control. This required a rapid and reproducible method of quantifying leaf size and shape parameter traits as well as serration characteristics. As was reported in [23], we were also interested to see how well PCA could be used to describe the variation in leaf area characteristics within this collection of trees.

## Implementation

**Figure 1:**
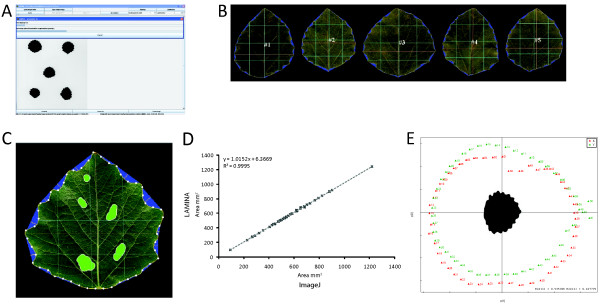
**Use of LAMINA to quantify leaf characteristics in the SwAsp collection**. **A **Screenshot of LAMINA. **B **Example cropped image generated by LAMINA showing dimension measurements and serration detection. **C **Example cropped image generated by LAMINA. Cavities (holes) in the leaf lamina are marked in green, serrations are marked in blue and the depth of each serration is marked by a yellow line. Horizontal and vertical centre lines are drawn in red with sub-divisions marked in blue. Boundary coordinates are shown as white circles along the perimeter. **D **Regression analysis to compare data generated from ImageJ to LAMINA for a set of 50 random images. **E **Principal Component Analysis loadings plot of X and Y coordinates generated for the SwAsp dataset using LAMINA (50 boundary coordinates per leaf). The leaf in the centre is the value closest to the centre of the cloud and has been oriented to match the distribution of XY values in the loadings plot. Component one appears to represent leaf width (55 % variance) and component two leaf length (27 % variance).

The LAMINA software has been implemented in Java as a stand-alone graphical application. The software is used to identify leaf objects and to calculate properties of those objects in an automated or semi-automated fashion. Automated analysis requires no user intervention after setting the desired parameters whereas semi-automated analysis pauses after each image has been analysed to allow manual adjustment of identified blade dimension centre lines (i.e. length and width), which can be important where leaves are not perpendicular to the image plane. An example screenshot of the user interface is shown in Figure 1A.

### Main computational steps

The computational processes involved can be described in the following sequential steps.

1. **Thresholding**. As an initial step, global thresholding is performed to find candidate picture elements (pixels) that putatively represent leaves. In the thresholding process, all pixel intensities are reduced from the typical grayscale range of 0–255 to either 0 (off; pixel is background) or 1 (on; pixel potentially belongs to a leaf object). As input, the inverse of the blue channel intensities are used rather than the entire RGB image. The rationale behind this strategy is that while leaves can be green, orange, red or even black, they are very rarely blue. On a white background, non-blue objects can, with high accuracy, be distinguished from the background using global thresholding.

The process of identifying a suitable global threshold can be performed either manually or automatically. In *manual thresholding*, the user specifies an arbitrary value *t *in the range 0–255, where pixels with intensities less than *t *will be set to 0 (background) and pixels with intensities equal to or greater than *t *will be set to 1 (putative leaves). The *automatic thresholding *procedure, on the other hand, tries to automatically determine a value of *t *that minimizes the variance of the thresholded image [26]. This procedure is generally suitable for images where objects have fairly well-behaved shapes, which is true for most leaf objects (see *Artemisia annua *section for an exception). The automated search procedure can be *greedy*, in which a local minima is found based on a greedy search starting from the mean value of the starting image. Alternatively, the procedure can be *exhaustive*, in which the entire range 0–255 is searched for the value *t *that minimizes the variance of the thresholded image. The latter procedure is generally more accurate but also considerably slower.

2. **Segmentation**. Posterior to thresholding, the input image has been reduced to a binary image containing pixels that are either background (0 = off) or potential leaf objects (1 = on). The task of segmentation is to group nearby pixels into segments (objects) that may potentially represent leaves. The segmentation starts by assigning an arbitrary on pixel as the current segment. The segment is then iteratively extended with neighbouring, unassigned on pixels (including diagonal pixels) until no more neighbouring on pixels exist. This procedure is repeated until all on pixels have been assigned to an object.

3. **Filtering**. Due to measurement noise and presence of contaminants in the image, some objects will not represent actual leaves. To remove dubious objects, filtering can be performed based on both the area of each object (to remove objects that are too small) and based on the density of each object (to remove e.g. black frames surrounding the image). The default filtering is non-stringent and will only remove the smallest objects, likely to represent contaminations in the image.

4. **Object boundaries**. The boundary of an object is defined by the set of on pixels where at least one neighbour of each on pixel is an off pixel (i.e. pixels on the surface of the object). The identification of the boundary pixels is a straightforward computational process. However, in order to simplify the subsequent steps, the adjacent boundary pixels are internally arranged sequentially (sorted) within each object. This procedure requires that distances are calculated between all boundary pixels and can be time-consuming for highly irregular surfaces, e.g. *Artemisia annua *images.

5. **Cavities**. Cavities in the leaf objects can be present due to e.g. biting herbivore damage, which implies that identification and measurement are of interest. A cavity is by definition surrounded by a boundary region that is unconnected to the outer boundary of the object. This distinctive characteristic is used to identify the cavities, seen as 'kinks' in the distances between neighboring boundary pixels. The off pixels that can only be connected to the cavity (inner) boundary define the cavity area. In this sense, cavities are defined as missing leaf area (holes) within the leaf lamina and do not account for herbivory starting at the edge of the leaf, which is computationally more difficult to quantify as it would require retrospective calculation of where the leaf boundary was previously. It is equally hard to distinguish herbivory or wounding at the leaf boundary from serrations. This represents an obvious area of future extension of LAMINA, but is not a trivial task.

6. **Serrations and indents**. Starting from a boundary pixel, the longest straight line that can be formed without crossing the object formed by non-boundary pixels is sought. The intermediate region between two serrations defines an indent. This is implemented in practice by connecting the starting boundary pixel with boundary pixels of increasing distances until a non-connectable boundary pixel is found. The last connectable pixel, i.e. one that can be connected by a straight line without crossing the object, is the next serration point. The process starts again using the latest serration point as the starting pixel. To allow for small variations in the boundary shape, a consecutive sequence of *k *non-connectable pixels are allowed before stopping. The parameter *k *can be adjusted by the user and determines the overall sensitivity of the serration identification algorithm.

7. **Indent depths**. Each indent is surrounded by two serrations that can be connected by a straight line. The indent depth is measured as the longest line to the base of the indent while being perpendicular to the straight line connecting the surrounding serrations. Due to the discrete nature of images, it is not always possible to achieve perfect perpendicularity, and hence a slight discrepancy in this angle is allowed.

8. **Boundary coordinates**. From the boundary pixels of each object, a fixed number of boundary coordinate points can optionally be identified. These are defined as equally spaced points around the surface of the object. The boundary coordinates are normalised against the centre coordinate of the object to make the measurements independent of the position of the object in the image.

### Output from LAMINA

After processing, LAMINA outputs cropped image files representing the identified objects after thresholding and segmentation. This allows the user to have a record of the results of the image analysis process (Figure 1B shows example cropped images within the LAMINA user iterface and Figure 1C an example generated cropped image). Furthermore, a number of quantitative measurements of the leaves are generated. This includes the leaf area, height, width, circularity, number of serrations, indent widths and depths as well as the boundary coordinates (normalised against leaf centre). For parameters that summarise several measurements, the output includes the mean, median and standard deviation.

### Scale calibration

Image measurements do not generally contain any information regarding the actual size of the image. In order to convert the pixel-based distances and areas in the leaf image into real quantitative measures, scale calibration has to be performed. The aim of the calibration is to determine the actual size of one pixel in millimeters (mm) and is optimally run once, to find the conversion ratio. LAMINA requires a calibration image to perform this calculation, containing one coloured object (not black) of known size on a white background. Ideally this object should fill the majority of the image area to maximize the accuracy of the calibration. After determining the measured pixel size of the image, and by manual input of the actual size in mm, the pixel-to-mm ratio can be determined and employed for all subsequent image calculations.

### Example applications of LAMINA

#### Exploring leaf physiognomy in the SwAsp Populus tremula collection

Full details of the common garden experiment can be found in [24]. Five leaves per replicate of each clone were sampled on a single day in early August 2007 into paper bags and later scanned using a Canon CanoScan 4400 F A4 flatbed scanner at a resolution of 300 dpi. A 40 × 50 mm yellow rectangle of card was scanned and used for scale calibration. Images were saved as jpeg files. The majority of genets (clones) were represented by four clonal replicates. Our sampling strategy was to select five random leaves from different heights on each replicate as we wanted to know how plastic (i.e. variable) leaf area was within and between both genets and intra-genet clonal replicates. The only criterion applied was that leaves should be mature and should not be from the terminal stem, as these leaves are of a fundamentally different nature in aspens. Images were analysed in LAMINA in a semi-automated work flow to allow for corrections to the orientation of leaves within the image. Default settings were used for all parameters except for the serration detection pixel threshold, where 22 was used. The centre line of each leaf was adjusted where required before proceeding to the next image. In total, 412 images containing 1879 leaves were analysed, with the LAMINA analysis taking 1 working day (8 h).

A random set of 50 leaves were scanned and analysed using ImageJ [20] and LAMINA. For the ImageJ analysis, images were imported as an image stack, at which point they were transformed to 8 bit (greyscale) and then thresholded using a value of 150 to produce a binary image. The *trace *tool was then used to select each leaf and the *Measure *tool used to record the selected area. The scale was set using the line tool to define a known distance using the same calibration image used for the LAMINA analysis. Data generated were analysed and visualised in R [27]. ANOVA tests were performed using the nlme package to test for clone within population and population effects. Principal Components Anlaysis (PCA) was performed in SIMCA P (v11.3, Umetrics, Sweden).

#### Benchmarking LAMINA using the complex leaves of Artemisia annua

*A. annua *leaves are highly complex and we deemed them to serve as a comprehensive test of the ability of LAMINA to extract and reliably quantify leaf area and dimension traits. We therefore undertook a more detailed method comparison using either glasshouse-grown or field-grown genotypes of *A. annua*. One mature leaf from six genotypes grown in a glasshouse was used to compare leaf area meter data to LAMINA. The area of each leaf was measured using a LI-COR LI-3100 Area Meter (LI-COR Environmental, Nebraska, USA) and the same leaves were scanned using an HP Scanjet 3570 c A4 flatbed scanner at 300 dpi. A 100 × 1 mm bar was scanned and used for scale calibration. Three mature leaves from 29 genotypes grown outside at Stockbridge Technology Centre, Cawood, North Yorkshire, U.K. were scanned using the same scanner and used for area analysis in LAMINA and ImageJ. Leaves 20, 21 and 22 (counting down from the top of the plant) were sampled in October 2007.

LAMINA analysis was performed using the following settings: Manual threshold value of 150, no serration detection.

ImageJ analysis was performed by transforming images to 8 bit (greyscale) and then thresholded using a value of 150 to produce a binary image. A polygon was then drawn around a leaf and the *Analyze Particles *tool used to calculate the area represented by leaf pixels. The scale was set by scanning a standard ruler and using the line tool to define a known distance. The use of the pixel analysis method, rather than the more automated method used for the aspen leaves, was required due to the complex shape of the *A. annua *leaves. However, this method increases the chance of any noise artifacts in the scanned image being included in the measurement calculations.

#### Testing LAMINA using species with diverse leaf shapes

In order to ensure that LAMINA functioned for a diverse range of species, we sampled leaves of a number of common European tree species as well as various poplar species and *A. thaliana*. One to three leaves per species were analysed to ensure that leaves were reliably extracted from the scanned images. All images were scanned as for the SwAsp trees. Additionally, the jpeg format images used as example applications in [23] and [21] were downloaded and analysed using LAMINA in order to benchmark our software against these other packages.

## Results and discussion

### Using LAMINA to explore leaf traits in the SwAsp collection

In order to test LAMINA and to provide us with an overview of leaf characteristics within the SwAsp collection to guide future experimental design, we sampled leaves from the northern common garden of the SwAsp collection [24]. As we had previously used ImageJ [20] for analysing leaf area, we first performed a comparison analysis between ImageJ and LAMINA as an initial benchmark to ensure that LAMINA provided comparable results. Both programs returned effectively identical measures of leaf area (Figure 1D), with an R^2 ^value of 0.99. Having established that LAMINA was functioning as intended, we then extended the analysis to the entire set of sampled leaves. Using this data, we first examined the variation between multiple leaves sampled from the same clonal replicate, which indicated that there was significant variation in leaf area within an individual plant (data not shown). This prompted us to extract only the leaf with the largest area measurement from each replicate, which reduced intra-genet variance, with the results shown in Table 1. The ANOVA model results in Table 1 show that, even after selecting only the largest leaf per replicate, there was still significant variance in leaf area within a genet. This result will be essential to guiding subsequent leaf sample collection and also indicates that very careful consideration should be given to sample collection not only for morphological analysis but also for other purposes such as physiology, transcriptomics and metabolomics, as leaf development appears to be highly plastic in aspen. We also examined the results of a PCA analysis of the boundary coordinate data and trait variable data produced by LAMINA. Figure 1E shows the loading plot of XY boundary coordinates for the set of data representing the largest leaf from each genet replicate. Both X and Y sets of coordinates form spherical distributions but they lie at right angles to each other. Principal component one appears to represent leaf width and component two leaf length, with these two components explaining the majority (82 %) of the variance in the data. PCA of the morphological trait values showed a distribution pattern confirming the correlation results shown in Table 1 (data not shown). We have therefore shown that LAMINA is suitable for extracting meaningful biological data using different PCA approaches in a fashion similar to [23] but with the additional advantage that traditional morphological measures of leaf traits are provided by LAMINA for use in methods other than PCA. The data produced by LAMINA is equally suitable for use in other analysis methods.

**Table 1:** Overview of leaf size and shape traits in the SwAsp trees

	**ANOVA**			
	Clone(Pop)	Population	Latitude	Longitude
Area	ns	ns	ns	ns
Length	ns	ns	ns	ns
Width	ns	**	ns	ns
Length:Width	ns	*	ns	ns
Circularity	ns	ns	*	ns
Horizontal symmetry	*	**	***	*
Vertical symmetry	ns	ns	ns	ns
Number of serrations	*	**	***	***
Indent depth	ns	ns	ns	ns
Indent width	ns	*	ns	*

ANOVA analysis of leaf size and shape parameter data generated using LAMINA. Significance values are * 0.05, ** 0.01, *** 0.001, ns not significant.

### Benchmarking LAMINA against ImageJ and a leaf area meter using Artemisia annua

Leaves from *A. annua *plants were by far the most complex in structure of those that we used for testing and developing LAMINA. We therefore examined the results generated for these leaves in more detail as a means of benchmarking LAMINA. Leaf area is an important trait in *A. annua *as this medicinal crop produces artemisinin, used in anti-malaria drugs, in glandular trichomes found predominantly on the leaf surface. Natural variation in *A. annua *leaves is being studied using QTL mapping and association studies, while induced mutations and phenotypes are being identified using forward and reverse genetic screens with all of these approaches requiring rapid and reliable quantification of leaf area.

**Figure 2:**
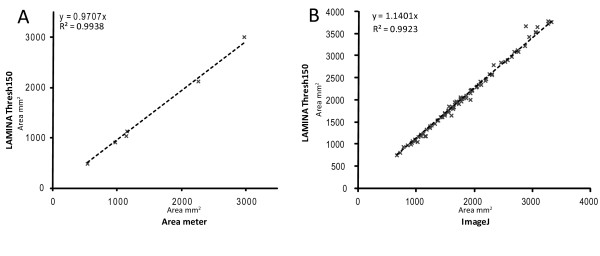
**Comparison of methods for quantifying leaf area in A. annua**. **A **Comparison of leaf area quantification using a leaf area meter and LAMINA. **B **Comparison of leaf area data generated using ImageJ and LAMINA.

We performed two small comparisons, one using a leaf area meter and the second using ImageJ as these two methods of calculating leaf area represent those most commonly used currently. Both methods provided effectively identical results for leaf area (Figure 2, R^2 ^= 0.9938 for the leaf area meter comparison and R^2 ^= 0.9923 for the ImageJ comparison). However, LAMINA has the added advantage of also providing a suite of additional measurements alongside area (although see below), as well as providing a far greater level of automation in the analysis pipeline. Both sets of results presented in Figure 2 show that LAMINA is able to reliably extract leaves from scanned images and accurately calculate leaf morphological traits from such complex leaves to a level of accuracy that matches existing, commonly-used analysis methods.

### LAMINA is suitable for use in a diverse range of species

**Figure 3:**
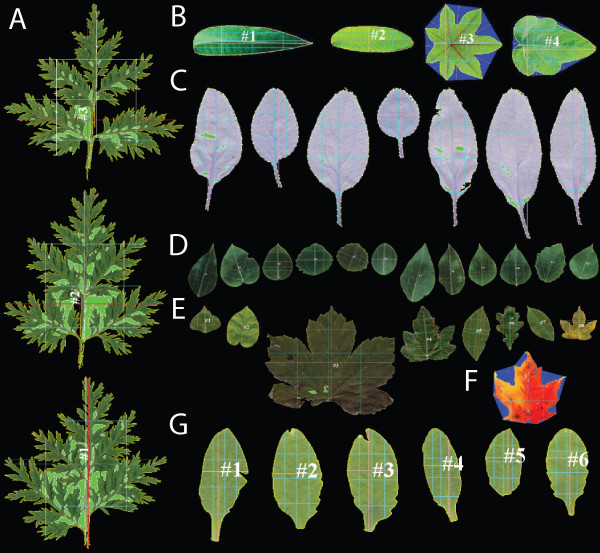
**Example cropped images generated using LAMINA in a range of species**. **A **Three example *Artemisia annua *leaves. Some regions are incorrectly identified as cavities, however the perimeter is correctly identified. **B **Example image from [21]. Serration detection pixel threshold = 50. **C **Example image from [23]. **D **Example *Populus *leaves from Umeå Plant Science Centre 2006 Calendar. **E **Example Image containing a range of leaves from common European tree species with contrasting leaf shapes. **F **Example use of serration detection to measure lobes in a senescing maple leaf. Serration detection pixel threshold = 75. **G **An example set of *Arabidopsis thaliana *leaves representing a developmental series. All images were analysed using the *Greedy search *threshold setting.

To qualitatively assess the general applicability of LAMINA, we scanned leaves from a diverse range of tree and flowering plant species. These included species commonly used in laboratory and genetics/ecology research as well as a range of species with divergent leaf shapes and forms. A number of examples of the cropped output images generated by LAMINA are shown in Figure 3, including the example images of [23] and [21], with the results showing that LAMINA performs equally well as existing software tools. We also tested LAMINA on a collection of scanned images of *Populus balsamifera *leaves, which have numerous, small serrations. LAMINA was able to quantify these small serrations provided image resolution was adequate (leaves were scanned at 600 dpi).

### Current limitations and future development

We have shown that LAMINA is able to accurately extract and quantify leaf area from scanned images of a diverse range of plant species. However, there are limitations to the use of the provided dimension measurements currently provided by LAMINA, and these limitations represent the most immediate targets for future development and expansion of LAMINA.

Although LAMINA is able to quantify leaf area of the *A. annua *leaves accurately, there are currently limitations to the use of additional measurements returned by LAMINA, with these being true in a range of leaf forms. Examining the *A. annua *leaves shown in Figure 3 shows that LAMINA currently returns blade dimensions using only straight lines, which is clearly far from ideal in these leaves. It is also clear that serration and cavity analysis will not return meaningful values from these leaves. There is therefore clear caution and consideration required by end users when making use of values returned by LAMINA. In the case of *A. annua*, we would suggest that calculated area is certainly reliable and that circularity may also be a useful indicator of how that leaf area is distributed. The use of any other returned values would require careful consideration by the end user.

As is the case for the *A. annua *leaves, many leaves do not have perfectly straight or symmetrical shapes and as such, the central line deviates from a straight line. Currently LAMINA only returns a measure of the maximum (or user set) straight line distance between the leaf base and tip. The inclusion of a tool to additionally allow manual placement of a non-straight line tracing the centre line (often the central vein) of a leaf is an obvious first target for extension of the current measures provided by LAMINA. At present, the difference between the values of the returned 25 % and 75 % vertical lengths can be used to indicate leaf asymmetry, which will often reflect the degree of leaf curvature and therefore the likely inaccuracy of the returned straight line centre measurement.

The *A. annua *leaves and the included example *A. thaliana *leaves shown in Figure 3 identify another important issue to consider when sampling leaves to be analysed using LAMINA – that of petioles: If petioles are sampled as well as actual leaf area, LAMINA will include the petiole as part of the leaf and this will affect generated measurements. In many species, removal of petioles is simple as there is a clearly identifiable boundary between leaf and petiole. If petioles are being removed, it is essential that this is done accurately as any remaining petiole will lead to the mis-identification of a serration either side of the remaining petiole. Figure 3G represents a more complex example, but one that is typical for many *A. thaliana *plants, where there is no clear boundary between the leaf lamina and the petiole. In such cases, it is often very hard to define where leaf becomes petiole and the sampling strategy must take this into consideration: LAMINA will include any scanned leaf area when generating dimension measurements and the end user must therefore decide what they wish to be included at the point of sampling (or by later manipulation of the generated scanned images to remove e.g. remaining, unwanted petiole area). Although it is not inconceivable that an algorithm could be developed to differentiate between leaf and petiole, this is certainly far from a trivial task, especially if such an algorithm should be generally applicable across species.

The example *A. thaliana *leaves in Figure 3G highlight another point that users must be aware of: currently, the software will not distinguish between wounding at the leaf boundary and serrations. In the cases shown in Figure 3, boundary damage most likely resulted from flattening the leaves at the point of image collection, as *A. thaliana *leaves are frequently curved and can not be flattened without tearing the leaf lamina. The use of median rather than mean serration values will limit the influence of such outliers but if serration quantification is being used, users should visually screen through the cropped images produced by LAMINA to identify problem leaves. It is possible that the algorithm for detecting leaf serrations could be extended to differentiate between boundary wounding or grazing herbivore damage (that typically extends from the boundary edge into the leaf) and actual serrations. However, as with other similar problems such as petiole detection, this will not be simple if the algorithm is to be applicable across a wide range of species (for example, many species contain serrations in combination with deeper, more infrequent, leaf lobes). Such algorithmic development would require extensive testing and confirmation across a broad range of species that have been exposed to a range of herbivore damage and wounding. There are also a number of potential extensions to LAMINA that we feel would have broad appeal to leaf researchers, including colour quantification (for example to track senescence), detection of necrotic lesions or flecks, measurement of leaf rust urediospore number and dimensions, and quantification of veinal pattern. As LAMINA has been released as an open source project using the well-supported Java language, it represents an ideal framework for the future integration of such extensions by the community and we hope that the instigation of such an open source project can serve as a means of concentrating development of a powerful phenotyping tool, as has been the case for the analysis of microscopy images since the initial release of ImageJ [20].

## Conclusion

We have developed a new software tool for the automated or semi-automated analysis of leaf morphological traits and have shown that the method is able to extract biologically meaningful data from a range of species with contrasting leaf shapes. The developed software performs equally well as existing software while also providing an extended range of measures of leaf size and shape indicators. We show that the software performs as well as commonly used leaf area meters, even when measuring highly complex leaf forms. Application of this software tool will significantly aid the rapid screening of large-scale collections of genotypes for forward or reverse genetics as well as equally serving plant breeders. This is the first open source tool available for the quantification of leaf serration.

## Availability and requirements

**Project name: LAMINA: **Leaf shApe deterMINAtion

**Project home page: **

**Operating system(s): **Platform independent

**Programming language: **Java

**Other requirements: **Java 1.4.x or higher. LAMINA uses the Java Advanced Imaging (JAI) package  to support common image file formats, which is bundled with the installation and hence no additional installation should be required.

**License: **GNU GPL2

## Abbreviations

LAMINA: Leaf shApe deterMINAtion; SwAsp: Swedish Aspen; QTL: Quantitative Trait Loci; PCA: Principal Component Analysis.

## Authors' contributions

MB developed the LAMINA software and contributed to the manuscript production. VS performed the *A. annua *leaf analysis and was supervised by AMR. RYS performed the SwAsp LAMINA analysis. JT, SJ, PG supervised the project. NRS conceived the project and tested the software, drafted the manuscript, scanned the SwAsp leaves and analysed the SwAsp leaf data. All authors read and approved the manuscript.
